# A pragmatic approach to the comparison of wrist-based cutpoints of physical activity intensity for the MotionWatch8 accelerometer in children

**DOI:** 10.1371/journal.pone.0234725

**Published:** 2020-06-19

**Authors:** Hsuan-Ping Lin, Nicole Lynk, Lynn L. Moore, Howard J. Cabral, Kevin S. Heffernan, Amy K. Dumas, Bryce Hruska, Rachel A. Zajdel, Brooks B. Gump, Nicole L. Spartano

**Affiliations:** 1 Department of Medicine/Preventative Medicine and Epidemiology, Boston University School of Medicine, Boston, MA, United States of America; 2 Department of Public Health, Food Studies, and Nutrition, Syracuse University, Syracuse, NY, United States of America; 3 Department of Biostatistics, Boston University School of Public Health, Boston, MA, United States of America; 4 Department of Exercise Science, Syracuse University, Syracuse, NY, United States of America; 5 Section of Endocrinology, Diabetes, Nutrition & Weight Management, Department of Medicine, Boston University School of Medicine, Boston, MA, United States of America; Indiana University, UNITED STATES

## Abstract

**Background:**

A variety of wearable monitors are available for objectively assessing physical activity but there is a lack of established values for the activity intensity of MotionWatch8 (MW8) and a similar lack of studies on comparability across devices. Our study aimed to establish activity intensity cutpoints for the MW8 accelerometer in children, which are necessary to determine whether they are meeting physical activity guidelines.

**Methods:**

Children (n = 39, ages 9–13 years) were asked to wear two different accelerometers (MW8 and ActiGraph) simultaneously on the same dominant wrist as they performed different activities designed to mimic activities of variable intensity that a child might perform in a free-living environment. Linear regression and receiver operating characteristic (ROC) curves were performed to assess sensitivity and specificity of the identified MW8 intensity cutpoints compared to established ActiGraph cutpoints.

**Results:**

Mean values for each activity were positively correlated using the MW8 and ActiGraph monitors (r = 0.85, p<0.001). The optimal cutpoints for differentiating sedentary from light physical activity, light from moderate, and moderate from vigorous activity were ≤32 counts, ≥ 371.5 counts, and ≥ 859.5 counts per 30 seconds, respectively.

**Conclusions:**

Our study demonstrated the ability of MW8 to discriminate different intensity activities and provided the first cutoff values for researchers using the MW8 to measure physical activity patterns among children.

## Introduction

Excess body weight in children is one of the most severe public health issues worldwide [1]. The consequences of childhood obesity entail both immediate and long-term health problems, such as increased risk of metabolic syndrome during youth and elevated long-term risk of cardiovascular disease in adulthood [2]. Therefore, childhood is the optimal time for early intervention aimed at preventing obesity and long-term health problems.

The American Heart Association (AHA) recommends that children achieve 60 minutes or more of moderate-to-vigorous aerobic activity every day and that this should include vigorous-intensity activity at least three days per week to decrease the risk of obesity and future cardiovascular disease [3]. Historically, epidemiologic studies have used self-reported measures due to the low cost and ease of administration [4]. However, a systematic review in 2009 demonstrated that 72% of self-reported instruments used in pediatric populations overestimated moderate-to-vigorous activity [5]. As such, better methods are needed for objective measurement of activity in children to allow for more accurate assessment of whether current guidelines are being met and to determine whether meeting the guidelines is associated with better health outcomes.

In the past decade, the number of accelerometer-based physical activity research publications has increased seven fold [6]. A systematic review, published in 2012, identified 40 different physical activity monitors that have been validated for use in the field [7], and many more have been validated since then. Unfortunately, the absolute count values from each device cannot be interpreted directly or compared with others because device manufacturers each use different methods to convert raw data into count values. Therefore, for every new accelerometer that becomes available, cutpoints should be established to allow the intensity of physical activity to be classified with accuracy [8]. It is also important that validation be tested in diverse populations across different age ranges.

The MotionWatch8 (MW8), is a wrist-worn, tri-axial, light-weight accelerometer (CamNtech Ltd, 2012), which can provide a long-term continuous recording of physical activity for up to six months without the need for recharging, comparing favorably with other accelerometers [9,10]. The MW8 has been validated in older adults as an objective measurement of physical activity [11]. However, to our knowledge, the MW8 has not been validated for measuring physical activity in children.

In our study, we tested the validity of the MW8 intensity cutpoints in children by performing a standardized set of activities using a pragmatic approach. We aimed to establish cutpoints for defining activity intensity in order to more readily compare the results of studies using the MW8 accelerometer to studies using the gold standard cutpoints set for ActiGraph devices, the most widely used commercially available instruments [9] that have been validated in children and adolescents [12,13].

## Methods

A total of 40 male and female participants (aged 9–13 years) from the Syracuse, New York community were enrolled in our study as part of the larger ongoing Environmental Exposures and Child Health Outcome (EECHO) study [14]. One participant was excluded from all analysis due to technical issues during monitoring, leaving 39 participants for analysis in the present study. The participants’ characteristics can be found in **Table 1**. All children were either African American or Caucasian without any serious medical or developmental disabilities based upon a health history questionnaire completed by their guardians. Participants who were on medications that may affect their cardiovascular system were asked to refrain from taking their medication on both visits of the study.

**Table 1:** Participant characteristics (N = 39).

Participant characteristics	Mean (SD) or N (%)
Age (years)	10.2 (1.2)
Height (inches)	57.2 (5.2)
Weight (pounds)	91.4 (34.8)
BMI (kg/m^2^)	19.1 (4.3)
BMI percentile (%)	59.7 (32.4)
Waist circumference (inches)	26.6 (4.7)
Gender	
Male	22 (56.4)
Female	17 (43.6)
Race	
Caucasian	27 (69.2)
African American	12 (30.8)
Pubertal development	
Pre-pubertal	6 (15.4)
Early puberty	8 (20.5)
Mid-puberty	19 (48.7)
Late puberty	6 (15.4)

Abbreviations: BMI, body mass index; SD, standard deviation.

All study procedures were approved by the institutional review board of Syracuse University. All guardians and participants provided written informed consent/assent prior to participating in the study. Children were asked to wear two accelerometers on their dominant wrist. They were then asked to perform a series of four activities designed to reflect different energy intensities that a child might perform in a free-living environment. The first activity was completed while participants were seated while completing oral consents and casual conversation with a researcher. Subsequent activities were conducted during a second visit in a gymnasium. For the second activity, participants were asked to walk to the beat of a slow-paced song (147 BPM song for four minutes). The third activity was a slightly faster-paced, 172 BPM song for another four minutes. Finally, during the fourth activity, children were instructed to play a game, moving balls from one container to another container as fast as possible for two minutes. The four activities were designed to straddle each intensity cutpoint, providing some intensity levels below, and some above the established intensity cutpoints. Our design enabled us to observe a wider range of activity intensities and thus test the accuracy of the MW8 device to discriminate among intensity levels in our study sample. Start and stop times were closely monitored by trial investigators and data were collected in 15-second epoch periods. To eliminate possible start/stop time discrepancies, the first 15 seconds and last 15 seconds for each activity were excluded from the analysis; remaining data were analyzed in consecutive 30-second increments, also to minimize the effect of discrepancies of a few seconds difference between device time capture. Due to time constraints of the study, only 20 children performed the first (resting) activity. All 40 children performed the second, third, and fourth activities.

The MW8 accelerometer (Actiwatch, CamNtech Ltd., Cambridge UK © 2012) is a tri-axial accelerometer designed to record acceleration ranging from 0.01-8g with a frequency range of 3–11 Hz, set to record at 15 second epochs. The 7 MW8 sensors used in our trial were new (factory calibrated) at the start of the trial. The ActiGraph model GT1M accelerometer (ActiGraph, LLC, Fort Walton Beach, FL) is a uniaxial accelerometer that records acceleration ranging of 0.05–2.5g with a frequency range of 0.25–2.5 Hz, set to record at 15 second epochs [15]. The ActiGraph intensity cutpoints derived for the GT3X model were chosen as the gold standard measures of physical activity intensity for our study because they have been extensively validated in children for measuring free-living physical activity [12]. Although a study comparing counts recorded by the GT1M and the GT3X find that they are not always consistent, the differences in counts did not impact classification of time spent in intensity categories [16], so the impact of using GT1M devices in the current study with cutpoints derived in GT3X devices is likely negligible. The GT1M ActiGraph devices we used for the current study were not new, we only used two for the duration of the trial and performed sensitivity analysis, observing no systematic differences in results using either sensor. The software used to produce activity counts for our study was MotionWare and ActiLife, respectively, for MotionWatch 8 and ActiGraph devices.

Factors that were considered as potential confounders included height, weight, BMI, and pubertal development. Height and weight were measured using a mechanical scale with a stadiometer (Detecto, Webb City, Missouri). To account for differences in growth and development, BMI percentile was used to express a participant’s BMI in relation to CDC growth charts.

Linear regression analysis was performed to evaluate the association between the activity recorded values by the validated ActiGraph monitor and the MW8 monitor. Receiver operator characteristic (ROC) analyses [17] were performed to determine optimal cutpoints for the MW8 associated with different intensity levels including sedentary, moderate-to-vigorous physical activity (MVPA), and vigorous activity compared with those previously established cutpoint values for the ActiGraph monitor in children by Crouter et al. using GT3X ActiGraph devices [13]. Previously-established cutpoints for the ActiGraph are as follows: sedentary activity: < 216 counts/30 seconds, MVPA: ≥ 2166 counts/30 seconds, and vigorous activity: ≥ 6780 counts/30 seconds, which should be comparable for our purposes to activity counts of the GT1M device used in the current study. Light activity is defined as the activity region between sedentary and MVPA counts/30 seconds. For the ROC curves, ActiGraph counts were coded as 0 or 1 according to the cutpoints established from Crouter et al [13]. For example, MW8 measurements were assigned “0” if they were recorded during time periods when the ActiGraph recorded counts < 2166 and “1” when ActiGraph recorded counts ≥ 2166.

The Youden index (*J*) was used to generate optimal cutpoints of MW8 for different levels of physical activity from the ROC curves, as previously described [18]. The sensitivity, specificity, and accuracy of each intensity cutpoint were expressed as area under the ROC curve (AUC) [19]. Sensitivity was defined as the probability that MW8 cutpoint correctly classified a given level of activity (true positive) whereas the specificity was the probability that MW8 cutpoint correctly identified those who were not at a particular intensity level [19]. In order to minimize false negatives and false positives equally, cutpoints for MVPA and vigorous activity were selected where the sum of the sensitivity and specificity was greatest. To choose the sedentary activity cutpoints, we decided to prioritize higher specificity to minimize the false positives which may occur due to arm movements.

All statistical analyses were performed with SPSS (version 24; IBM, Somers, NY) and SAS studio (version 3.6; SAS Institute Inc, Cary, NC). A significance level of alpha = 0.05 was used to indicate statistical significance. All values are reported as mean ± standard deviation (SD).

## Results

The 39 children in the study had a mean age of 10.2 ± 1.2 years; 43.6% were female, and had an average BMI percentile = 59.7% ± 32.4% (**Table 1**). Our study sample was bi-racial, with 30.8% of participants being African American and 69.2% Caucasian children, and a wide range of pubertal status. Study characteristics for the subsample of participants that performed the sitting/resting activity are provided in **Table 2** (n = 19).

**Table 2:** Characteristics of participants who performed the resting activity (N = 19).

Participant characteristic	Mean (SD) or N (%)
Age (years)	9.8 (0.9)
Height (inches)	56 (4.6)
Weight (pounds)	83.1 (26.5)
BMI (kg/m^2^)	18.4 (3.9)
BMI percentile (%)	54.8 (32.6)
Waist circumference (inches)	25.9 (4.5)
Gender	
Male	12 (63.2)
Female	7 (36.8)
Race	
Caucasian	12 (63.2)
African American	7 (36.8)
Pubertal development	
Pre-pubertal	1 (5.3)
Early puberty	6 (31.6)
Mid-puberty	10 (52.6)
Late puberty	2 (10.5)

**Fig 1:**
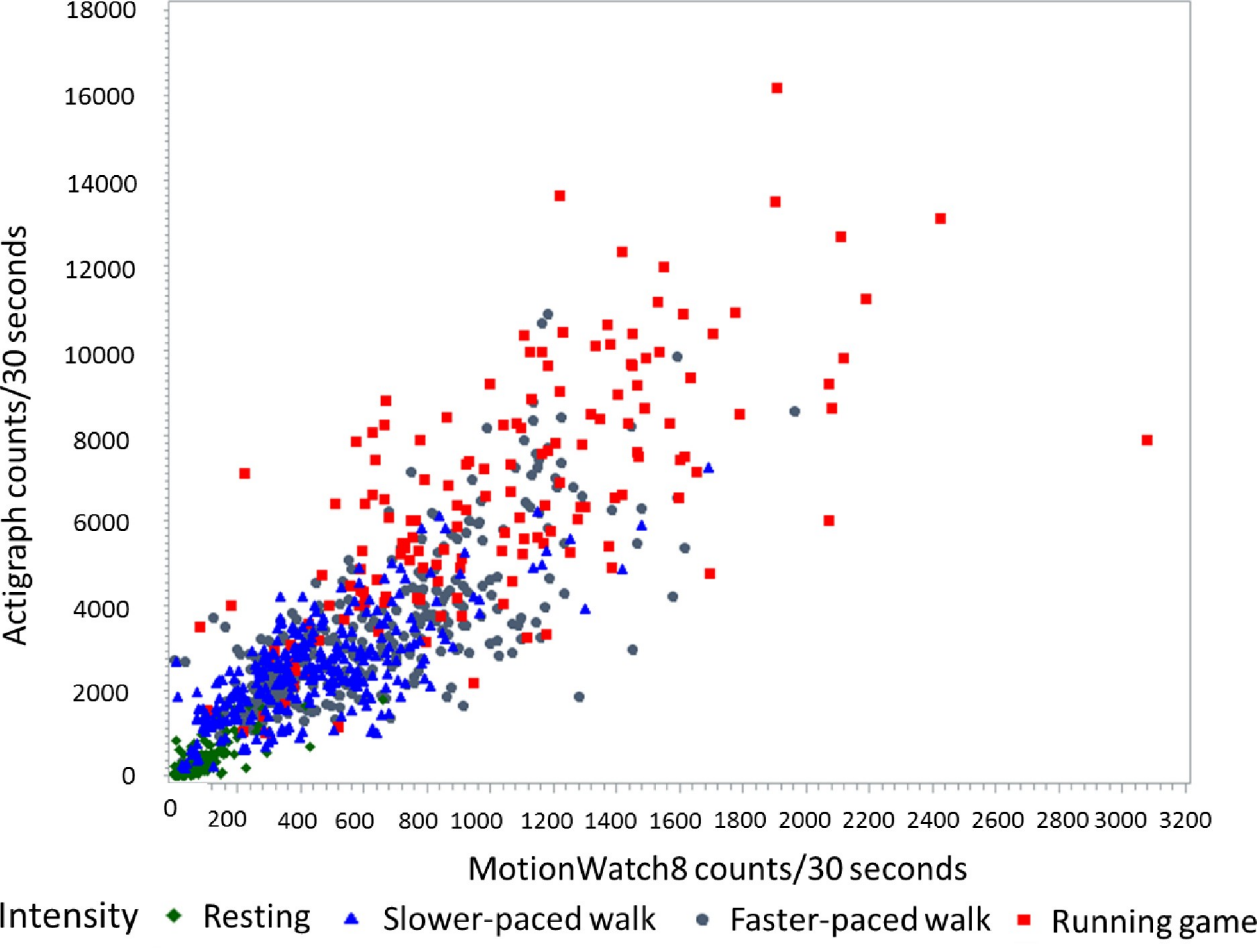
Correlation between mean output from the MotionWatch8 and Actigraph accelerometers. The scatterplot demonstrates higher absolute counts of motion (acceleration) measured using the Actigraph compared to the MotionWatch8, but positive correlation (r = 0.85, p<0.001) between devices when worn in the same participants simultaneously. Each study participant contributed three or four points on the scatterplot to represent four different intensities of movement during a structured activity (Diamond: resting, triangle: slower-paced walk, dot: faster-walk, square: running game). Only 13 of the total 39 participants contributed data for the resting activity.

The mean ActiGraph and MW8 accelerometer counts for every 30 seconds are shown in **Table 3** for each of four prescribed activities—(1) sitting/resting, (2) slower-pace walking, (3) faster paced walking, and (4) running game. Average counts/30 second intervals for the MW8 were strongly, positively correlated with average counts/30 second from the ActiGraph (r = 0.85, p<0.001) (**Fig 1**). For both accelerometers, there was a large increase in counts/30 seconds between the sitting/resting activity and the slower-paced walk. The rate of increase in the counts/30 seconds from each activity level to the next was much higher for the ActiGraph device than for the MW8 device (**Table 3**).

**Table 3:** Mean (SD) counts/30 seconds for prescribed activities measured with ActiGraph and MotionWatch8.

Activity	N	ActiGraph counts/30 sec	MotionWatch8 counts/30 sec
1. Sitting/Resting	19	293 (272)	69 (63)
2. Slower-paced walk (147 BPM song)	39	2481 (948)	435 (230)
3. Faster-paced walk (172 BPM song)	39	3420 (1402)	630 (300)
4. Running game	39	6569 (2278)	1040 (417)

**Fig 2:**
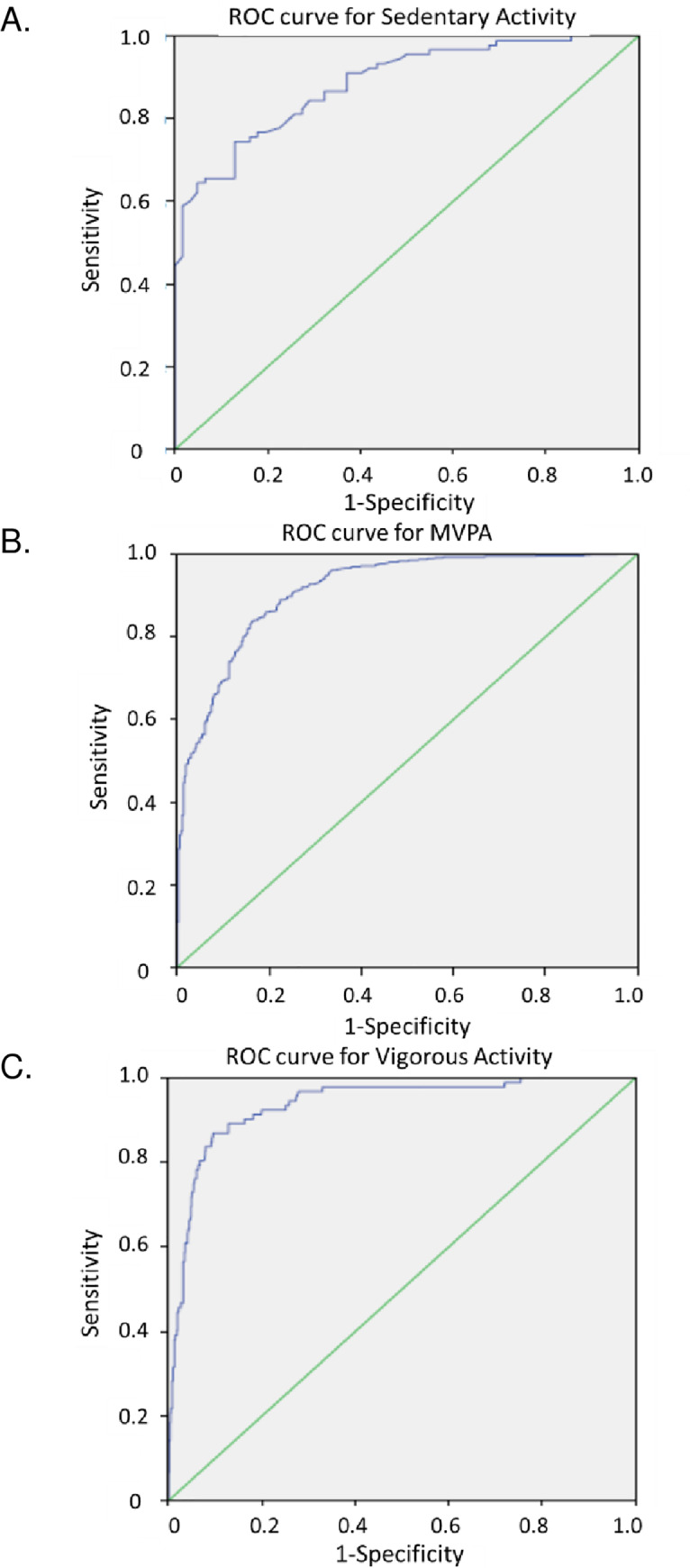
Receiver operator characteristic (ROC) curves for activities of different intensities. Sensitivity and specificity were defined at different MotionWatch cutpoints for agreement with the intensity level defined by Actigraph (using previously established cutpoints) for the following activity intensities: A) sedentary activity, B) Moderate-to-vigorous physical activity (MVPA), and C) Vigorous activity.

ROC curves were created to identify cutpoints for sedentary, moderate-to-vigorous, and vigorous physical activity (**Fig 2A through 2C**). The area under the curve (AUC) was used to evaluate classification accuracy for different intensities of physical activity. The MW8 activity intensity cutpoints that we established to distinguish vigorous from non-vigorous activity, and MVPA from non-MVPA both performed well (AUC: 0.94, 95% CI: 0.91, 0.96. AUC: 0.89, 95% CI: 0.84, 0.94, respectively), while the AUC for sedentary activity was the least precise at 0.89 (95% CI: 0.84, 0.94) (**Table 4**).

**Table 4:** Selected cutpoints and corresponding sensitivity and specificity values for MotionWatch8 per 30 seconds.

	Cutpoints, counts/30 seconds	Sample size for ROC analysis	Sensitivity	Specificity	Area under ROC curve (AUC)
Sedentary	≤32	19	0.70	0.87	0.89
Light^a^	32.5–371	-	-	-	
MVPA	≥371.5	39	0.84	0.84	0.91
Moderate^b^	371.5–859	-	-	-	
Vigorous	≥859.5	39	0.89	0.87	0.94

Abbreviations: MVPA, moderate to vigorous activity.

^a^ Light activity is the range between are the boundaries for sedentary activity and MVPA.

^b^ Moderate activity is the range between the lowest level for MVPA and cutoff value for vigorous activity.

The values of MW8 cutpoints, sensitivity, and specificity for sedentary, MVPA, and vigorous activities are listed in **Table 4**. The Youden index (*J*) was used to generate optimal cutpoints. We selected our cutpoint for sedentary behavior by optimizing specificity to avoid incorrectly classifying light or moderate intensity of physical activity as sedentary behavior. Our approach yielded a cutpoint for sedentary behavior of ≤32 counts/30seconds with a sensitivity level of 70% and specificity of 87%. In order to minimize false negatives and false positives equally, cutpoints for MVPA and vigorous activity were selected in which the sum of the sensitivity and specificity was greatest. The cutpoint chosen for MVPA was ≥ 371.5 counts/30seconds, which yielded a sensitivity and specificity of 84%. The cutpoint for vigorous activity was ≥ 859.5 counts/30seconds, which yielded a sensitivity of 89% and specificity of 87%. The cutpoints for light activity are defined as the region between the boundaries for sedentary activity and MVPA. The cutpoint for moderate activity is at the boundaries for MVPA and vigorous activity.

A previous MW8 validation study in older adults by Landry et al. [11] suggested that the optimal cutpoints for sedentary and MVPA were ≤ 89.25 and ≥ 281.25 counts/30seconds, respectively. By applying the cutpoints to our study, both sedentary behavior and MVPA yielded higher sensitivity, but lower specificity (91% vs. 59% and 94% vs. 69%, respectively, data not shown).

## Discussion

The primary purpose of our study was to establish the optimal cutpoints of wrist-based accelerometer MW8 for the classification of sedentary, moderate-to-vigorous, and vigorous activity in children to provide an accurate method for assessing the intensity of physical activity. We, first, observed that the values recorded simultaneously by the MW8 device and the ActiGraph device during the activities were strongly correlated. Next, we identified optimal cutpoints to differentiate sedentary from light physical activity, light from MVPA, and moderate from vigorous activity (≤32 counts, ≥ 371.5 counts, and ≥ 859.5 counts per 30 seconds, respectively).

Childhood is an ideal window for early lifestyle interventions aimed at preventing obesity and future risk of metabolic syndrome and cardiovascular disease [2]. Due to feasibility and cost, the most commonly used tool to assess physical activity in children is self-report or proxy-report from parents [20]. Pedometers and accelerometers have been increasingly popular due to their ability to reduce the bias inherent to self-reported data; they can also be used in large population-based studies [21]. However, pedometers do not provide precise measures of total daily movement, particularly since pedometers cannot detect all types of activity. The accelerometer with multiple axis measurements, by contrast, can better capture total body movement and also distinguish the intensity of physical activity [22].

The placement of a given accelerometer on the body impacts validity of the physical activity assessment [23]. Positioning the monitor on the hip, close to the center of the body, better reflects whole-body movement, thus yielding higher correlations with energy expenditure [24]. However, certain activities mainly require considerable upper-body movement and these activities range from playing computer games, basketball or racquet sports, or using a punching bag; such activities may not be precisely measured using hip-mounted accelerometers [25]. A recent statement from the Kaiser Family Foundation (using data from 2009) reported that 11–14 year old children spend more time playing computer and video games (3 hours 11 minutes per day) than younger or older children (1 hour 47 minutes and 2 hours 47 minutes, respectively, per day) [26]. Moreover, using wrist-based accelerometers for measuring activity have been reported to have much better compliance than hip-mounted accelerometers [27]. Therefore, choosing wrist-based accelerometers may overcome the low compliance issue and potentially capture more upper-body movement. Of course, one concern with using wrist-based accelerometers is that they may underestimate sedentary time because wrist motions occurring during sedentary activities may lead to misclassification. Our study identified cutpoints for a wrist-based accelerometer, MW8, for classifying physical activity intensity among children, but caution must be applied when interpreting sedentary time thresholds, given the limitations of wrist-based placement of these devices.

To our knowledge, there has been only one study in older adults aimed at identifying activity intensity cutpoints with the MW8 [11]. We chose to examine the sensitivity, specificity, and accuracy (expressed as the AUC) of the activity intensity cutpoints identified in this group of children using the MW8 compared with a previously-validated ActiGraph device intensity cutpoints. Our cutpoints for defining sedentary activity in this group of children were lower than those for older adults in the study by Landry et al. [11]. The AUC for sedentary activity from the study of older adults was 0.81 while the AUC in our study of children for the same activity level was 0.89. In the older adult study, the AUC for MVPA was 0.79 while in our study of children, the AUC for our cutpoints was 0.91. AUC scores above 0.9 are considered to have excellent accuracy. Scores from 0.80–0.89 are considered good, 0.70–0.79 are considered fair, and those below 0.70 to have poor accuracy [19]. Thus the MW8 performance in children in the current study ranged from was generally good to excellent.

There are several factors that may contribute to the differences in cutpoints between children and adults. First, intensity cutpoints from the previous study were established in older adults (aged 57–80 years) while current study examined children (aged 9–13 years). Comparable activities among children are generally less efficient than the same activity performed by an adult [28], which may lead to different count values for a similar activity performed by children and adults. Secondly, we selected 30-second time intervals, instead of the 1-minute intervals used in the previous study [11]. Our shorter time periods may have allowed us to capture the variable patterns of MVPA and vigorous activities for children [12]. Considering the various intensity cutpoints we established compared with the previous study, it is clear that different intensity cutpoints are likely needed for population groups of different ages.

Another issue associated with measuring habitual activity patterns in children is how to accurately define sedentary behavior. Sedentary activity is difficult to identify by any device due to challenges in differentiating between sitting and standing [29]. Johansson et al. demonstrated that the time spent in sedentary behavior recorded by wrist-based accelerometers was overestimated when maximizing the specificity and sensitivity of ROC analysis in preschool children [30]. Kim et al. also indicated that sedentary time is often overestimated in school-aged children when applying established cutpoints for accelerometers to classify sedentary behavior [25]. We sought to avoid this problem by prioritizing specificity of sedentary cutpoints, which may prevent light activity from being misclassified as sedentary behavior.

This study has several limitations. Firstly, the intensity cutpoints in this study were determined predominantly in a laboratory setting. Moreover, only four different activities were included in the study that may not fully mimic the daily activity a child might perform in a free-living environment. The findings should be further confirmed in free-living conditions over multiple days. Secondly, due to the transient movements of children’s behavior, a recording time interval shorter than 30 seconds may need to be examined in future studies. Moreover, because of the challenges that can accompany establishing sedentary cutpoints, utilizing other methods such as a daily log may complement the MW8 to identify sedentary behavior. Lastly, we did not use indirect calorimetry, the true gold standard method for measuring energy expenditure [31], to assess physical activity intensity cutpoints. Instead, we applied intensity cutpoints from the ActiGraph (established from indirect calorimetry validation studies) to estimate energy expenditure, which may introduce some error into our validation methods. The use of intensity cutpoints established in GT3X devices for categorization of activities measured by GT1M devices may also contribute to some measurement error, but likely does not impact the categorization of activities into intensity levels by much, according to authors of a previous validation paper [16]. Additionally, our intensity cutpoints may only be suitable for healthy children, given that physical conditions may affect energy expenditure [32]. Future works should conduct larger validation trials using MW8 devices (as well as other accelerometers), aimed at evaluating whether different cutpoints should be used for different populations, based on age, sex, height, or body composition.

## Conclusion

To our knowledge, our study is the first study evaluating physical activity intensity cutpoints, including cutpoints for sedentary activity, for the MW8 in children [9]. Given the increasing need for lifestyle interventions during childhood [2], these intensity cutpoints for the MW8 provide researchers with an objective tool for recording habitual patterns of activity intensity in children.
